# Acute fatty liver of pregnancy cases in a maternal and child health hospital of China: Three case reports

**DOI:** 10.1097/MD.0000000000021110

**Published:** 2020-07-17

**Authors:** Ling Wang, Quan Gan, Shuguo Du, Yun Zhao, Guoqiang Sun, Ying Lin, Ruyan Li

**Affiliations:** a Department of Obstetrics; b Department of Intensive Care Unit, Maternal and Child Health Hospital of Hubei Province, Tongji Medical College, Huazhong University of Science and Technology. Wuhan, China.

**Keywords:** acute fatty liver of pregnancy, misdiagnosis, postpartum hemorrhage

## Abstract

**Rationale:**

Acute fatty liver of pregnancy (AFLP) is extremely hazardous to pregnant woman in the 3rd trimester of pregnancy. AFLP has an insidious onset and nonspecific experimental indicators, which therefore is difficult to be diagnosed.

**Patient concerns:**

Case 1 was transferred to our hospital for hypertensive disorders complicating pregnancy at gestation of 38 weeks + 3 days. Case 2 was transferred to our hospital for suspicious fetal heart monitoring response at gestation of 36 weeks + 4 days. Case 3 was transferred to our hospital for prelabor rupture of membranes at gestation of 37 weeks + 1 days.

**Diagnosis:**

The diagnosis of AFLP was based on the Swansea criteria.

**Interventions:**

All 3 cases were delivered by cesarean section, and they were all transferred to intensive care unit for further treatment. Cases 2 and 3 were subjected to plasma exchange and continuous renal replacement therapy.

**Outcomes:**

In this study, all 3 patients were initially diagnosed as gastritis. In addition, case 1 was diagnosed as preeclampsia and her AFLP was misdiagnosed with postpartum hemorrhage after cesarean delivery. Case 2 was admitted to the hospital for intrahepatic cholestasis of pregnancy and fetal distress, but we considered it as AFLP before delivery. Case 3 was treated according to severe intrahepatic cholestasis of pregnancy, but we rediagnosed it as postpartum hemorrhage and disseminated intravascular coagulation after cesarean delivery. Neonatal asphyxia and complications were not found. All of the 3 cases were fully recovered and discharged from our hospital.

**Lessons:**

If there are multiple risk factors including vomiting, abdominal pain, and fetal distress, AFLP should be highly suspected. Early diagnosis, especially before termination of pregnancy, is the key to successful treatment of AFLP.

## 1 Introduction

In 1940, Sheehan for the 1st time described acute fatty liver of pregnancy (AFLP) as a specific clinical entity,^[1]^ but the etiology and pathogenesis of such disease remains unclear. Maternal death in AFLP is associated with complications such as disseminated intravascular coagulation, hepatic encephalopathy, and acute renal failure.^[2,3]^ These serious conditions usually occur in the 3rd trimester or in the immediate postpartum period.^[4]^ Women with AFLP are recommended to treated in a center with expertises including high-risk obstetrics, maternal-fetal medicine, neonatology, and hepatology. If necessary, they are recommended to be monitored in the intensive care unit (ICU).^[4]^

## 2 Methods

This study presents a retrospective case review of 3 cases of AFLP patients admitted from January 1, 2019 to June 1, 2019 in a Maternal and Child Health Hospital of Hubei province, Tongji Medical College, Huazhong University of Science and Technology, China.

Clinical data were obtained from the medical records reviewing, including maternal age, gestational age at admission, parity and gravidity, fetal sex, fetal number, Apgar score, clinical manifestation, laboratory tests, hospitalization time, and maternal-perinatal outcome. Ethical approval was not thought to be necessary because this is a retrospective study and all the data were dealt only with the patients’ medical records and follow-up results via telephone and WeChat. All included women signed informed consent for therapeutic procedures and also for the publication of this case report.

In this study, the diagnosis of AFLP was based on the Swansea criteria.^[5]^ A pregnant patient will be diagnosed with AFLP if she shows 6 (or more) out of following 14 criteria: vomiting, abdominal pain, polydipsia/polyuria, encephalopathy, elevated bilirubin, bilirubin >0.8 mg/dL, hypoglycemia <72 mg/dL, elevated uric acid >950 mg/dL, leukocytosis >11 × 10^9^/L, ascites, alanine aminotransferase >42 IU/L, ammonia >66 μmol, renal impairment, acute kidney injury or Cr >1.7 mg/dL, coagulopathy or prothrombin time >14 seconds, and microvesicular steatosis on liver biopsy. Liver biopsy is typically characterized by liver cell swelling, vacuolation, and pallor during filtration, accompanied by microvesicular fatty liver. The 3 cases in this study were in compliance with this diagnostic criterion. However, the Swansea criteria are meant to be applied to cases with no other liver diseases of pregnancy, such as HELLP syndrome; hemolysis, elevated Liver enzymes, Low plateiet coullt (HELLP).

Ethical approval was not thought to be necessary because this is a retrospective study and all the data were obtained by the patients’ medical records and follow-up results via telephone and WeChat. All included pregnant women signed informed consent for therapeutic procedures and also for the publication of this case report.

## 3 Case reports

### 3.1 Case 1

A 29-year-old pregnant woman, in the 1st pregnancy, was transferred to our hospital for hypertensive disorder complicating pregnancy at gestation of 38 weeks + 3 days. On admission, the patient had no headache, vomiting or other symptoms. Cesarean section was performed a few minutes later after admission to deal with fetal distress. The operation proceeded smoothly, but postpartum hemorrhage occurred 156 minutes later. In the meantime, obvious abnormalities of liver and kidney function were reported by laboratory. Therefore, the medical history was immediately questioned again. As the patient recalled, she had nausea, vomiting, and upper abdominal discomfort a week ago, and then went to the internal medicine department, treated as gastritis. However, she did not take it seriously and had not told us in advance. Only then did we consider acute fatty liver. We applied comprehensive treatment such as the use of double balloon catheter, blood transfusion, and liver protection to stabilize the patient's condition. Subsequently, the patient was transferred to ICU for further treatment. After 10 days, she was recovered and discharged from hospital. The newborn was male, with birth weight of 2400 g, which was small for gestational age, but Apgar score was full marks, so the newborn was not transferred to neonatal intensive care unit (NICU).

### 3.2 Case 2

A 28-year-old pregnant woman, in the 1st pregnancy, was transferred to our hospital for suspicious fetal heart monitoring response at gestation of 36 weeks + 4 days. On admission, the patient already had symptoms including nausea, vomiting, and upper abdominal for 5 days. She had been treated as gastritis. To confirm the diagnosis, we conducted a physical examination for the patient and found jaundice. Soon the biochemical results revealed significant abnormalities of liver and kidney function as well as slight abnormality of coagulation function. We had a consultation and considered it as AFLP. After adequate preoperative preparation, cesarean section was proceeded smoothly. The operative indication was fetal distress. Because of preoperative anemia and coagulation dysfunction, blood products were transfused immediately after delivery. All these efforts eventually led to the avoidance of postpartum hemorrhage. This patient was transferred to ICU for 10 days of comprehensive treatment, including plasma exchange (PE) and continuous renal replacement therapy, eventually recovered well. The newborn was male, with birth weight of 2475 g, and full marks in Apgar score. The newborn was transferred to neonatal department due to premature delivery for 11 days of observation and treatment.

### 3.3 Case 3

A 32-year-old pregnant woman, in the 2nd pregnancy (para 0, 1 early abortion), was transferred to our hospital for prelabor rupture of membranes at gestation of 37 weeks + 1 days. On admission, the patient had nausea and jaundice. Like cases 1 and 2, she had been diagnosed and treated as gastritis. Cesarean section was performed to deal with fetal distress. The operation went smoothly, but postpartum hemorrhage occurred 91 minutes after operation, vaginal bleeding, and oozing from the wound observed. According to clinical symptoms, abnormal coagulation function and abnormal liver and kidney function, we considered it as acute fatty liver. Therapeutically, we started with stitching up the wound to stop the bleeding, and transfused blood products at the same time to improve coagulation function. The volume of postpartum hemorrhage was about 1800 mL. The patient was transferred to ICU for further treatment. The symptoms, signs, and examination results all suggested that the patient's condition was the heaviest of the 3 cases. Then we applied PE and continuous renal replacement therapy twice to improve the clinical symptoms and laboratory characteristics. The recovery time of this case was also the longest and the length of hospitalization lasted for 11 days. Like cases 1 and 2, the newborn was male, with birth weight of 2330 g, and Apgar score was 9/1 minutes; 10/5 minutes. Being younger than the actual gestational age, the newborn was transferred to neonatal department for 11 days of observation and treatment.

The clinical and obstetrical features of 3 patients are shown in Table 1, and the management schemes of 3 cases during hospitalization are shown in Table 2.

**Table 1:**
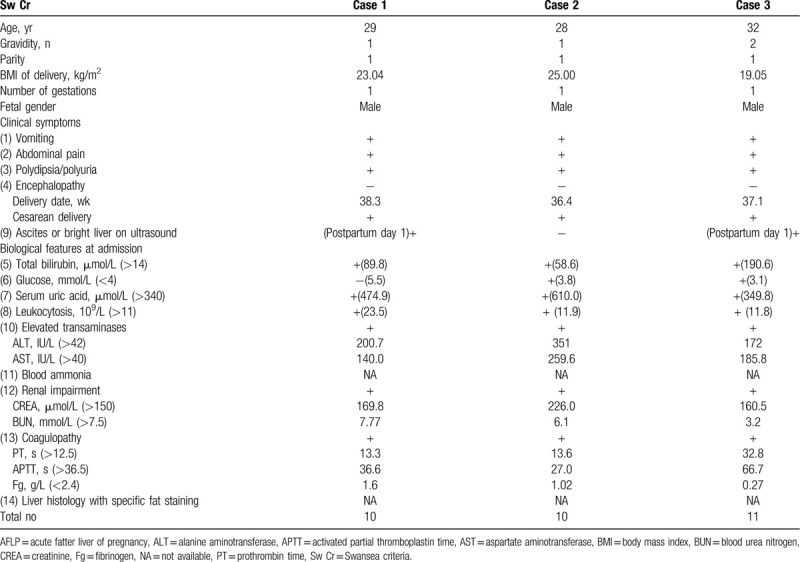
Clinical and obstetrical features of 3 patients affected by AFLP.

**Table 2:**
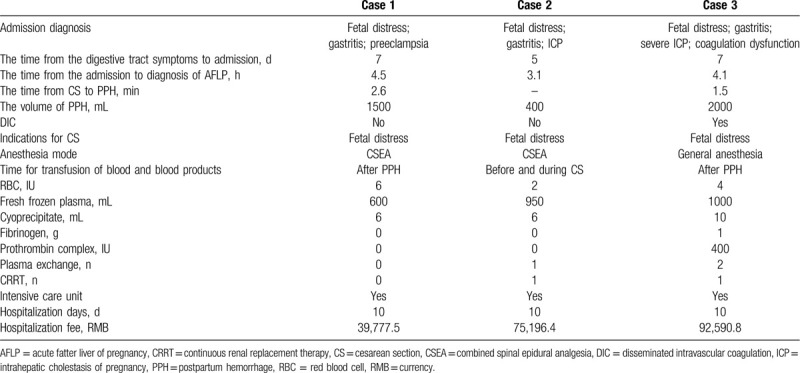
Management in cases with AFLP during hospitalization.

## 4 Discussion

The AFLP is a rare but life-threatening complication especially in the 3rd trimester of pregnancy (median presentation 36 weeks).^[6]^ The etiology and pathogenesis of AFLP is still unknown. Diagnosis of AFLP is mainly based on clinical symptoms and laboratory findings. AFLP has an insidious onset and nonspecific experimental indicators, which therefore is usually difficult to be diagnosed accurately. There are no uniform diagnostic criteria or guidelines on this disease in the world. The current common diagnostic criteria are Swansea criteria mentioned earlier.

In the early stage, AFLP patients normally show atypical symptoms such as nausea, vomiting, and vague abdominal pain. According to another study by Nelson et al involving 51 AFLP patients (1975–2012), the most common complaints were nausea and vomiting (57%), hypertension (57%), and abdominal pain (53%).^[7]^ Therefore, it is easy to be ignored or misdiagnosed as gastroenteritis, acute severe hepatitis, and so on.^[8]^ In this study, all 3 cases were misdiagnosed as gastritis. A definite diagnosis of AFLP cannot be confirmed until laboratory results or unexplained postpartum hemorrhage occurs. We were surprised to find that the 2nd case had less bleeding, which was attributed to the timely diagnosis of AFLP before cesarean delivery. Also, we did adequate preparation before the operation, such as blood transfusion which was used to improve coagulation function. Ultimately, postpartum hemorrhage and other complications were avoided. It is frequently happened that the 1st-visit physician did not recognize the importance of the onset of nausea and vomiting in the 3rd trimester. Therefore, education of midwives and junior doctors should be strengthened to emphasize that nausea and vomiting in 3rd trimester may a sign of possible AFLP. The most important lesson learned from these cases is that early recognition and diagnosis are the pivotal factors in the management of AFLP.

Risk factors for AFLP include primipara, male sex of the fetus, previous episode of AFLP, and coexisting diagnosis of other liver disorders of pregnancy such as HELLP syndrome.^[9]^ Higher body mass index is also found to be associated with AFLP.^[10]^ Two or 3 risk factors can be identified for each case. To prevent serious complications, we must conduct intensive monitoring for high-risk factors and improve the quality of medical care. Hirotada Suzuki reported that the soluble fms-like tyrosinekinase 1/placental growth factor ratio may be used to rapidly distinguish AFLP from HELLP syndrome.^[11]^ More researches are needed to find a way to predict AFLP. Through these case studies, we conclude that if there are multiple risk factors, AFLP should be highly suspected and treated accordingly. Waiting for confirming of all symptoms may delay the treatment.

The successful treatment of the cases cannot be separated from timely induction of labor. AFLP is harmful to both mothers and infants. Another lesson learned from these cases is to terminate pregnancy in time to avoid adverse maternal and child outcomes. However, there is a lack of guidelines on selecting the delivery mode for women with AFLP. In recent years, some researchers have recommended the use of cesarean section to improve the fetal prognosis, and caesarean section is proved to be the safest method of delivery, which should be recommended to lower the risk of adverse pregnancy outcomes in AFLP.^[12,13]^

Moreover, the management of AFLP is a multidisciplinary progress that necessitates active and prompt intervention by intensive care physicians and obstetricians along with anesthesiologists and neonatologists in a tertiary care center equipped with an ICU. The successful treatment of the 3 cases is inseparable from multidisciplinary cooperation. Therapeutic PE has emerged as a life-saving approach, which has been demonstrated to be effective in treatment of acute liver failure.^[14]^ Artificial liver support system is one of the effective methods for the treatment of liver failure.^[15]^ In these cases, no maternal deaths or severe encephalopathy were reported. This may be due to the effectiveness of PE therapy or the relatively mild condition of the patient.

Neonatal mortality induced by AFLP has been reported to be about 15% to 66%.^[16]^ It was fortunate that all 3 newborns were in good condition, which might be benefited from timely termination of pregnancy and appropriate treatment in neonatology. Special attention must be paid to fetal heart rate monitoring as it often shows no response or frequent deceleration. In this study, we found abnormal fetal heart rate and used it as a surgical indication to avoid adverse outcomes in newborns. It was found that amniotic fluid was polluted by meconium, and continuing to wait might lead to adverse outcomes.

In summary, early diagnosis and timely termination of pregnancy are the keys to successful treatment of AFLP. At the same time, it should be noted that the management of AFLP is a multidisciplinary progress.

## Acknowledgments

The authors are grateful to the patients who gave their informed consent for publication. They also thank Dr Quan Gan for guiding the combination treatment of AFLP.

## Author contributions

**Conceptualization:** Ling Wang, Quan Gan, Shuguo Du, Yun Zhao, Guoqiang Sun, Ying Lin, Ruyan Li.

**Data curation:** Ling Wang, Quan Gan, Shuguo Du, Yun Zhao.

**Formal analysis:** Ling Wang.

**Investigation:** Ling Wang, Yun Zhao.

**Methodology:** Ling Wang, Quan Gan, Yun Zhao.

**Project administration:** Shuguo Du, Yun Zhao, Guoqiang Sun, Ying Lin, Ruyan Li.

**Supervision:** Ling Wang.

**Validation:** Ling Wang, Quan Gan, Yun Zhao.

**Writing – original draft:** Ling Wang, Yun Zhao.

**Writing – review & editing:** Ling Wang, Quan Gan, Shuguo Du, Yun Zhao, Guoqiang Sun, Ying Lin, Ruyan Li.
